# Aquaporin 4 regulation by ginsenoside Rb1 intervenes with oxygen-glucose deprivation/reoxygenation-induced astrocyte injury

**DOI:** 10.1097/MD.0000000000017591

**Published:** 2019-10-18

**Authors:** Ya-Nan Li, Zhong-Wen Gao, Ran Li, Yun-Feng Zhang, Qing-San Zhu, Fei Huang

**Affiliations:** a Department of Pediatrics, The First Hospital of Jilin University; b Department of Molecular Biology, Basic Medical College of Jilin University; c Department of Orthopedics, China-Japan Union Hospital of Jilin University, Changchun, Jilin, PR China.

**Keywords:** AQP4, ginsenoside Rb1, oxygen-glucose deprivation/reoxygenation, spinal cord ischemia-reperfusion injury

## Abstract

**Background:**

Spinal cord ischemia-reperfusion injury (SCII) is a common complication of spinal surgery as well as thoracic and abdominal surgery. Acute cytotoxic edema is the key pathogenic alteration. Therefore, avoiding or decreasing cellular edema has become the major target for SCII treatment.

**Methods:**

The antiedema activity of ginsenoside Rb1 on aquaporin (AQP) 4, nerve growth factor (NGF), and brain-derived neurotrophic factor expression was detected by western blot and real-time polymerase chain reaction under conditions of oxygen-glucose deprivation/reoxygenation (OGD/R) in a rat astrocyte model in vitro. In addition, the cellular membrane permeability of AQP4 overexpressing cells or AQP4 small interfering RNA-transfected cells was detected.

**Results:**

Ginsenoside Rb1 significantly prevented OGD/R-induced AQP4 downregulation in rat astrocytes. In addition, ginsenoside Rb1 treatment or AQP4 overexpression in rat astrocytes significantly attenuated the OGD/R-induced increase of cellular membrane permeability. Moreover, ginsenoside Rb1 obviously prevented the OGD/R-induced decrease of NGF and BDNT expression in rat astrocytes.

**Conclusion:**

These findings demonstrate that ginsenoside Rb1 can relieve spinal cord edema and improve neurological function by increasing AQP4 expression.

## 1 Introduction

Spinal cord ischemia-reperfusion injury (SCII) is a common complication of spinal surgery as well as thoracic and abdominal surgery. It causes paralysis, heavy mental stress, and economic burdens to society and the patient's family. Most SCII patients are young and middle-aged laborers, and the mortality rate is as high as 3.8% to 7.6%.^[1]^ Although the pathogenesis of SCII is not fully understood, acute cytotoxic edema at the injured site is the key pathogenic alteration, which could be used to determine the prognosis of SCII.^[2]^ Therefore, avoiding or decreasing cell edema has become the major target for SCII treatment.

Aquaporin (AQP), a water channel protein, plays a crucial role in the maintenance of water balance and the internal environment. Thirteen AQPs have been discovered so far, including AQP0 to AQP12.^[3,4]^ Among them, AQP4 plays an important role in the transmembrane transport of water molecules. Abnormal expression of AQP4 can result in pathological edema.^[5,6]^ AQP4 is mainly distributed in astrocytes in the spinal cord and brain. To maintain the water balance in the central nervous system, AQP4 mainly processes water uptake and water transfer at the end foot of astrocytes.^[7]^ Although the related research is still limited, it is clear that the abnormal expression of AQP4 in brain astrocytes is closely related to cerebral edema formation.^[8]^ It is noteworthy that the cellular composition of the spinal cord is similar to that of the brain, and the pathogenesis of edema in SCII is similar to that of brain edema. Therefore, it is speculated that the pathogenesis of edema in SCII is related to the abnormal expression of AQP4.

Ginsenoside Rb1 monomer (molecular formula: C_54_H_92_O_23_, molecular weight: 1109.29 Da) is a traditional Chinese medicine ginseng extract. Ginsenoside monomer has biological activities of antioxidation, antiapoptosis, and nerve protection.^[9,10]^ In recent years, Chinese scholars have tried to use ginsenoside monomer for the treatment of nervous system diseases and nerve injury.

To date, there are no effective drugs for the treatment of SCII available in the clinic. The drugs most frequently used for SCII management are glucocorticoids, nerve growth factor (NGF), and neurotrophic factor-3, which can reduce spinal cord edema to various degrees. However, their drawbacks of a limited efficacy, high side effects, and a high cost are unsatisfactory to clinicians.^[11–13]^ Therefore, it is important to develop new drugs for SCII treatment, especially amelioration of spinal cord neuronal edema. Ginsenosides are a group of steroid glycosides and triterpene saponin natural products that are widely used in traditional Chinese medicine.^[14]^ In this study, we explored the role and possible signaling pathways of ginsenoside Rb1 in AQP4 induction in rat astrocytes in vitro. The results of this study could provide a new method for the clinical prevention and treatment of SCII edema.

## 2 Materials and Methods

### 2.1 Primary astrocyte culture and oxygen-glucose deprivation/reperfusion (OGD/R) model

Primary astrocytes were prepared from the myeloid tissues of neonatal rats, according to a method described previously.^[15]^ The prepared cells were cultured in glucose-free Dulbecco modified Eagle medium (DMEM; Gibco, Rockville, MD) in a 37°C incubator containing a humidified gas mixture of 1% O_2_/95% N_2_/4% CO_2_ for 2 hours. For reoxygenation, the cells were incubated for 24 hours with normal DMEM containing 10% fetal calf serum in a 37°C incubator containing humidified 5% CO_2_/95% air. The OGD/R model was established as described previously^[16]^ to simulate the SCII process. The experimental protocols were approved by the Jilin University Animal Ethics Committee

### 2.2 Cellular survival assay by the 3-(4,5-dimethylthiazol-2-yl)-2,5-diphenyltetrazolium bromide (MTT) assay

The cytotoxicity of ginsenoside Rb1 (purity ≥98.00%; Sigma-Aldrich, St. Louis, MO) in astrocytes was determined by measuring the cellular viability using the colorimetric MTT assay (Promega, Madison, WI). Briefly, starved astrocytes (10^5^ cells/well) were cultured at 37°C and 5% CO_2_ for 72 hours in a 96-well plate in the absence or presence of various concentrations of ginsenoside Rb1 (32.00 μg/mL, 25.60 μg/mL, 20.48 μg/mL, 16.38 μg/mL, 13.12 μg/mL, 10.49 μg/mL, 8.39 μg/mL, or 6.71 μg/mL, respectively; each reaction condition was replicated in 4 wells). After staining with 0.5 mg/mL MTT for 4 hours under 5% CO_2_ at 37°C in an incubator, the absorbance at 570 nm was measured by a plate reader (BioTek, Winooski, VT). Each test was repeated twice. The optical density value of the nondrug-treated cells was normalized as a 100% survival rate. A ginsenoside Rb1 dose of 16.38 μg/mL provided a survival rate of 97.26%; therefore, it was defined as the maximal tolerable concentration.

### 2.3 Western blot

Western blot was employed to evaluate AQP4 protein expression in astrocytes. Total protein extract (50 μg) was loaded onto a 10% sodium dodecyl sulphate-polyacrylamide gel, separated by electrophoresis, and transferred onto a nitrocellulose membrane (Sartorius, Goettingen, Germany). After blocking with 5% milk for 1 hours, the membrane was incubated with polyclonal AQP4 (1:500, ab46182) or β-actin (1:1,000; ab8227) antibody (Abcam, Cambridge, UK) overnight at 4°C and then horseradish peroxidase-conjugated goat anti-rabbit polyclonal IgG (1:800; sc2004; Santa Cruz Biotechnology, Inc, Dallas, TX) for 1 hours at room temperature. The probed target proteins were then visualized with enhanced chemiluminescence reagent (Santa Cruz Biotechnology, Inc) and exposed on an X-ray film.

### 2.4 Real-time reverse transcription-polymerase chain reaction (RT-PCR)

Total RNA from astrocytes was extracted by using TRIzol reagent (Invitrogen, Carlsbad, CA), according to the manufacturer's instructions. The corresponding complementary DNA was synthesized by a reverse transcription kit (Promega), and quantitation of the target gene expression was evaluated by real-time RT-PCR with SYBR Green (Bio-Rad, Richmond, California), according to the manufacturer's instructions. The human AQP4 primers were as follows: sense, 5′-ATTGGGAGTCACCACGGTTCAT-3′; antisense, 5′-TGGATTCATGCTGGCTCCGGTAT-3′. The β-actin primers were as follows: sense, 5′-TCACCCACACTGTGCCCATCTACG-3′; antisense, 5′-GGATGCCACAGGATTCCATACCCA-3′. A total of 40 amplification cycles (94°C for 30 seconds, 58°C for 30 seconds, and 72°C for 40 seconds) were performed. The results were analyzed using the 2^−ΔΔCT^ method.^[17]^

### 2.5 Gene clone, small interfering RNA (siRNA), and gene transfection

The rat AQP4 siRNA oligonucleotide (5′-GCCAAGTGGAGACAGAAGA-3′) and negative control sequence (5′-TTCTCCGAACGTGTCACGT-3′) were purchased from Gene-Pharma (Suzhou, China). For the gene expression study, the full-length sequence (972 bp) of the rat *AQP4* gene was synthesized by PCR and cloned into the LV5 vector at the *Not I* and Nsi I restriction sites by GENEWIZ Biology Company (Beijing, China).

Astrocytes (1 × 10^6^) were cultured in a 6-well culture plate with complete DMEM/high-glucose medium (Gibco, Grand Island, NY) containing 10% fetal bovine serum (PAA, Pasching, Austria) and 1% penicillin/streptomycin in an incubator at 37°C with a humidified atmosphere and 5% CO_2_. The complete DMEM medium was replaced by Opti-MEM serum-free medium (Gibco) when the cell fusion level reached more than 80%.

The AQP4-LV5 plasmid or AQP4 siRNA was transfected into the rat astrocytes by using Polybrene Transfection Reagent (Sigma-Aldrich, St. Louis, MO). Briefly, the mixture of AQP4-LV5 plasmid or AQP4 siRNA and polybrene (6 μg/mL) was prepared at a 100:1 ratio and transfected into astrocytes, according to the manufacturer's instructions. After 24 hours, the transfection medium was replaced with complete DMEM, and these cells were used for the subsequent experiments.

### 2.6 Enzyme-linked immunosorbent assay (ELISA)

The expression levels of NGF and brain-derived neurotrophic factor (BDNF) in the supernatants of the coculture medium were determined with the corresponding commercial ELISA kits (eBioscience, San Diego, CA), according to the manufacturer's instructions.

### 2.7 Astrocyte volume changes and osmotic water permeability

The cellular water permeability was detected by the hypotonic medium-induced water influx method, according to the description of Capurro et al^[18]^ Astrocyte volumes were examined by a fluorescence–self-quenching method with calcein-AM dye (Sigma-Aldrich). Briefly, astrocytes were incubated with 150 mOsm hypotonic phosphate-buffered saline (PBS) for 30 minutes and stained with 2 mM calcein-AM for 30 minutes. The calcein-loaded astrocytes were then mounted on glass slides, and the images were captured by an inverted epifluorescence microscope equipped with a computer-controlled charge-coupled device camera (Sony Corp, Tokyo, Japan). The cellular volume of the captured images was analyzed by ImageJ software (http://imagej.nih.gov/ij/).

### 2.8 In vitro experiments

To evaluate the role of AQP4 on the neuroprotective activity of ginsenoside Rb1 in vitro, we first intervened with AQP4 messenger RNA (mRNA) expression in the astrocytes by *AQP4* gene transfection or AQP4 silencing. The astrocytes were then assigned to the following groups: control, OGD/R, OGD/R + AQP4 overexpression, OGD/R + AQP4 overexpression-vector, OGD/R + AQP4 overexpression + ginsenoside Rb1, OGD/R + si-AQP4, OGD/R + si-vector, and OGD/R + si-AQP4 + ginsenoside Rb1. The *AQP4* gene expression and silencing were evaluated by real-time PCR and western blot analyses. Meanwhile, the effects of *AQP4* gene silencing on the cell water permeability were determined as well. Next, the astrocytes were treated with ginsenoside Rb1 and divided into the following 4 groups: control, OGD/R, OGD/R + PBS, and OGD/R + ginsenoside Rb1. The effects of ginsenoside Rb1 on astrocytes were analyzed by *AQP4* gene expression, functional detection, cytokine analysis, and water permeability examination.

### 2.9 Statistical analysis

All data are presented as the mean ± standard deviation. The differences between the control and test groups were calculated by the analysis of variance test. *P* < .05 was considered statistically significant.

## 3 Results

### 3.1 The maximum tolerable concentration of ginsenoside Rb1 in astrocytes

**Figure 1:**
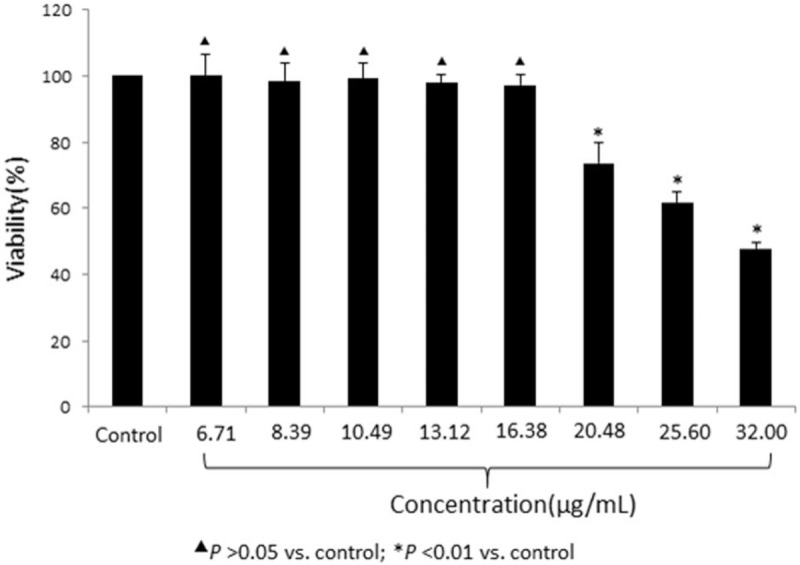
Cellular toxic effect of ginsenoside Rb1 on rat astrocytes by the MTT assay. Rat astrocytes were treated with the indicated concentration of ginsenoside Rb1, and the cellular viability was detected by the MTT assay. The viability of the control (PBS treatment) group was normalized as 100%. Each data point represents the mean of 4 independent assays. ^▴^*P* > .05 versus the control group; ^∗^*P* < .01 versus the control group. MTT = 3-(4,5-dimethylthiazol-2-yl)-2,5-diphenyltetrazolium bromide, PBS = phosphate-buffered saline.

The maximum tolerable concentration of ginsenoside Rb1 was examined by the MTT assay. After treatment with different concentrations of ginsenoside Rb1 (32.00 μg/mL, 25.60 μg/mL, 20.48 μg/mL, 16.38 μg/mL, 13.12 μg/mL, 10.49 μg/mL, 8.39 μg/mL, or 6.71 μg/mL, respectively) for 72 hours, the MTT results showed that a ginsenoside Rb1 concentration less than or equal to 16.38 μg/mL produced no appreciable effects on astrocyte viability (*P* < .05). Thus, the maximum tolerable concentration of ginsenoside Rb1 for the cultured astrocytes was 16.38 μg/mL (Fig. 1).

### 3.2 Regulation of AQP4 protein expression by ginsenoside Rb1 in astrocytes

**Figure 2:**
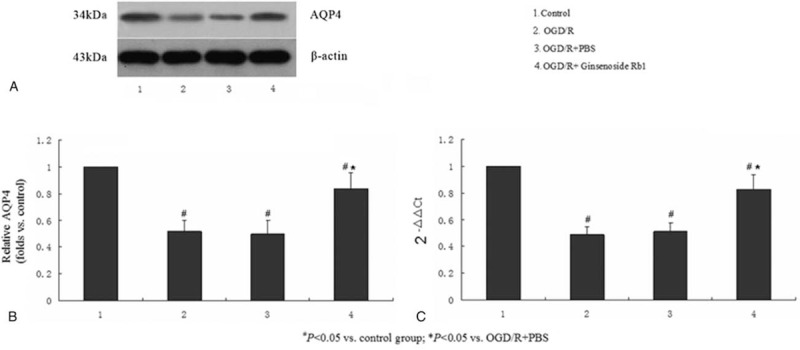
Ginsenoside Rb1 prevented the OGD/R-induced AQP4 downregulation in astrocytes. Western blot and real-time PCR analysis of AQP4 protein (A and B) and mRNA (C) expression in the astrocytes treated with the OGD/R model, PBS in the OGD/R model, and ginsenoside Rb1 in the OGD/R model. Nontreatment (control) was used as a negative control. Each data point represents the mean of 3 independent assays. ^#^*P* < .05 versus the control group; ^∗^*P* < .05 versus the OGD/R+PBS group. AQP4 = aquaporin-4, mRNA = messenger RNA, OGD/R = oxygen-glucose deprivation/reperfusion, PBS = phosphate-buffered saline, PCR = polymerase chain reaction.

To determine the effect of ginsenoside Rb1 on the expression of AQP4 in astrocytes, the OGD/R model was constructed, and the cells were divided into 4 groups (control, OGD/R, OGD/R + PBS, and OGD/R + ginsenoside Rb1). The AQP4 protein expression levels in the astrocytes of the OGD/R, OGD/R + PBS, and OGD/R + ginsenoside Rb1 groups were significantly less than that of the control group (*P* < .05). Moreover, the AQP4 protein expression of the OGD/R + ginsenoside Rb1 group was significantly greater than that of the OGD/R + PBS group (*P* < .05) (Fig. 2A and B). A similar response pattern was observed at the gene expression level by real-time PCR (Fig. 2B). These results indicated that ginsenoside Rb1 significantly prevented the OGD-induced AQP4 expression decrease in astrocytes and that AQP4 mediates the antiedema activity of ginsenoside Rb1.

### 3.3 *AQP4* gene silencing efficiency

**Figure 3:**
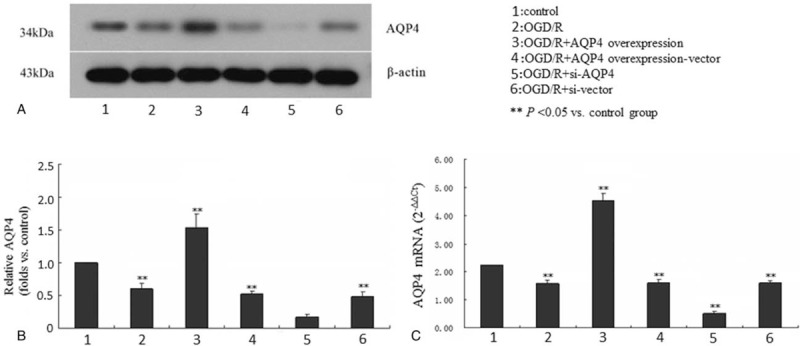
*AQP4* gene expression interference by siRNA and overexpression in astrocytes. Astrocytes were transfected with AQP4-LV5 or AQP4 siRNA. The AQP4 protein (A and B) and mRNA (C) expression were analyzed by western blot and real-time RT-PCR. Nontreatment (control), OGD/R, LV5 vector (overexpression vector), and siRNA control (scrambled) sequence were used as controls. A typical representative western blot image is shown in A. Quantification of the western blot and real-time PCR results are presented in B and C, respectively. Each data point represents the mean of 3 independent assays. ^∗∗^*P* < .05 versus the control group. AQP4 = aquaporin-4, mRNA = messenger RNA, OGD/R = oxygen-glucose deprivation/reperfusion, RT-PCR = real-time reverse transcription-polymerase chain reaction, siRNA = small interfering RNA.

To further investigate the crucial role of AQP4 in the edema process, we established *AQP4* gene knockdown by the corresponding siRNA as well as AQP4-overexpression models in astrocytes. Figure 3 shows the AQP4 protein (Fig. 3A and B) and mRNA (Fig. 3C) expression levels in the AQP4-silenced and -overexpressed cells. The AQP4 protein expression in the OGD/R model was significantly less than that of the control group; these findings were similar to those shown in Figure 2. On the other hand, the AQP4 protein expression was clearly elevated by AQP4-LV5 transfection (OGD/R + AQP4), compared with the vector transfection only group (OGD/R + AQP4-overexpression vector). Next, the AQP4 protein expression was obviously decreased after AQP4-siRNA transfection (OGD/R + si-AQP4), compared with the vector-transfected control (OGD/R + si-vector). A similar pattern was obtained for the AQP4 mRNA expression as detected by real-time PCR (Fig. 3C).

### 3.4 Ginsenoside Rb1 attenuated OGD-induced cellular water permeability in astrocytes

**Figure 4:**
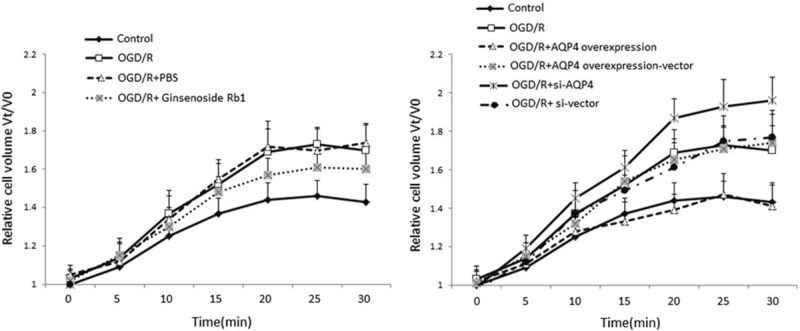
Ginsenoside Rb1 and AQP4 prevented the OGD/R-induced cellular membrane water permeability increases in astrocytes. The effect of OGD/R and ginsenoside Rb1 on cellular membrane water permeability was detected under the indicated conditions in astrocytes (A). Furthermore, similar detection was performed in the AQP4-overexpressed or AQP4-knockdown (B) astrocytes. Each data point represents the mean of 3 independent assays. AQP4 = aquaporin-4, OGD/R = oxygen-glucose deprivation/reperfusion.

The cellular edema was estimated by a cellular membrane water permeability assay to verify whether ginsenoside Rb1 could prevent OGD-induced cellular edema. The results showed that the exposure of astrocytes to hypotonic solution for 20 to 30 minutes significantly elevated the astrocyte water permeability compared with the normal control, suggesting that OGD/R can increase cellular water permeability to induce cellular edema. However, ginsenoside Rb1 treatment obviously decreased the OGD-induced water permeability elevation (Fig. 4A).

### 3.5 Antiedema potential of AQP4 in astrocytes

The results shown in Figures 2 and 3 indicate that AQP4 expression was significantly decreased in the OGD cellular model in astrocytes, suggesting that AQP4 might have antiedema potential and play an important role in the antiedema activity of ginsenoside Rb1. Next, we knocked down the *AQP4* gene with the corresponding siRNA to see whether *AQP4* gene silencing could induce cellular edema by a cellular membrane water permeability assay. For this purpose, we measured the time course of astrocyte swelling (*Vt*/*V*0) in response to a hypotonic solution. Interesting, *AQP4* gene silencing (astrocytes + si-AQP4) significantly increased the astrocyte cellular water permeability at 15 to 30 minutes after exposure to hypotonic solution (Fig. 4B). Meanwhile, *AQP4* gene overexpression (astrocytes + AQP4 overexpression) significantly decreased the astrocyte cellular water permeability at 15 to 30 minutes after exposure to a hypotonic solution (Fig. 4B). Furthermore, ginsenoside Rb1 did not alleviate astrocyte swelling caused by *AQP4* gene silencing. These data suggest that ginsenoside Rb1 does have antiedema potential and that the antiedema activity is via AQP4. At the same time, ginsenoside Rb1 did not affect the cellular water permeability of astrocytes with AQP4 overexpression. It is speculated that AQP4 overexpression restored the cellular water permeability to normal; therefore, ginsenoside Rb1 did not further affect the water permeability in the astrocytes. These results indicate that ginsenoside Rb1 can only restore the water permeability of astrocytes to normal, but not overcorrect it to excessive levels, so as to protect the astrocytes.

### 3.6 Ginsenoside Rb1 partially attenuated the OGD-induced decrease of NGF and BDNF expression in astrocytes

**Figure 5:**
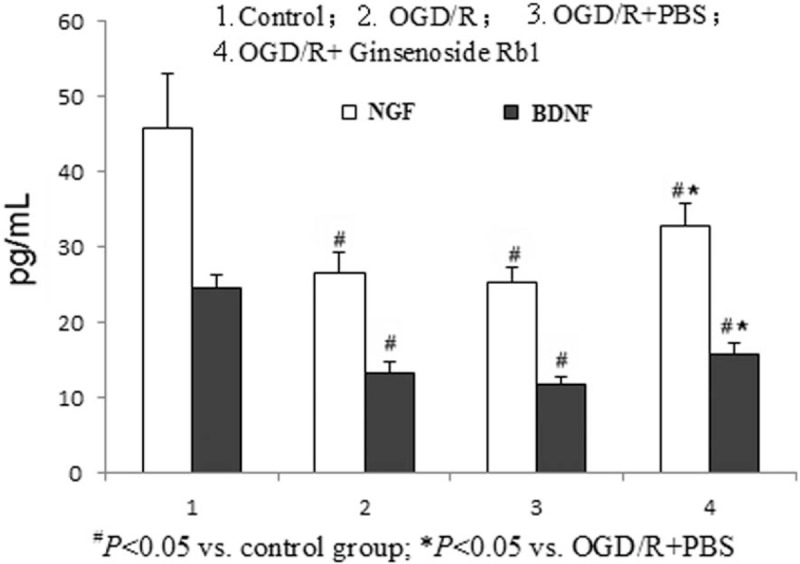
Ginsenoside Rb1 attenuated the OGD/R-induced NGF and BDNF downregulation in astrocytes. The protein expression of NGF (white) and BDNF (black) was detected in astrocytes under the indicated conditions. Each data point represents the mean of 3 independent assays. ^#^*P* < .05 versus control; ^★^*P* < .05 versus OGD/R + PBS; ^▴^*P* < .05 versus OGD/R + AQP4 overexpression; ^☆^*P* < .05 versus OGD/R + si-AQP4. AQP4 = aquaporin-4, BDNF = brain-derived neurotrophic factor, NGF = nerve growth factor, OGD/R = oxygen-glucose deprivation/reperfusion.

NGF has been reported to attenuate AQP4-induced edema in rats.^[19]^ BDNF is a member of the neurotrophin family, which participates in the protection of certain neurons of the peripheral and central nervous systems.^[20]^ Here, we investigated whether NGF and BDNF are involved in OGD-induced astrocyte edema as well as the mechanism of ginsenoside-Rb1 reducing edema. The expression levels of NGF and BDNF in cellular culture medium were detected by ELISA in the different treatment groups as detailed above. The results showed that the expression levels of NGF and BDNF in the OGD/R and OGD/R + PBS groups were significantly less than those in the control group (*P* < .05), and there was no significant difference between the 2 groups. Compared with the OGD/R + PBS group, ginsenoside Rb1 obviously increased the expression of NGF and BDNF (*P* < .05), but the expression levels were still significantly less than those of the control group (*P* < .05) (Fig. 5). Furthermore, compared with OGD/R, AQP4 silencing or overexpression did not affect the expression of NGF or BDNF, and ginsenoside Rb1 could significantly increase NGF and BDNF expression in the astrocytes with either AQP4 silencing or overexpression. These results indicated that NGF and BDNF were downregulated during the edema process (OGD model) and that ginsenoside Rb1 can partially reverse the OGD-induced downregulation of NGF and BDNF expression in astrocytes. Moreover, NGF and BDNF may be the upstream regulators of AQP4. Thus, the silencing or overexpression of AQP4 does not significantly stimulate the expression of NGF or BDNF.

## 4 Discussion

Spinal cord edema is a serious evident pathophysiological change in SCII.^[21]^ During the SCII process, a large number of vasoactive substances are produced, leading to increased capillary permeability, which leads to spinal edema.^[21]^ AQP4 widely expressed in the nervous system and plays viral role in water transport. In particular, a negative correlation between AQP4 and spinal cord edema has been recently reported.^[22]^ Currently, the most widely recognized model that can simulate ischemia-reperfusion injury at the cellular level is the oxygen-glucose deprivation/reoxygenation (OGD/R).^[23]^ In spinal cord tissues, AQP4 is expressed most in astrocytes, and the latter play an indispensable role in the function of spinal cord.^[22]^ Therefore, the astrocyte OGD/R model can be used to simulate the microenvironment of SCII in vitro. In this study, we determined that AQP4 expression was downregulated in the astrocyte OGD model of ischemia and reperfusion (Fig. 2), suggesting that AQP4 may play a pivotal role during the SCII process. This finding further confirms our previous result that AQP4 is downregulated in SCII.^[24]^ In addition, Zhang et al have reported that miR-130b is an upstream regulator that controls AQP4 expression in an astrocyte OGD model.^[25]^ Thus, whether miR-130b participates in the pathological process of SCII would be an interesting topic for further study.

Based on the above data, we believe that increasing the expression of AQP4 in vivo could alleviate the edema caused by SCII. Our previous study showed that SCII in a mouse model resulted in the reduced expression of AQP4 in the spinal cord, which could gradually recover over time. Furthermore, ginsenoside Rb1 treatment significantly attenuated the AQP4 decrease to protect the integrity of the astrocytes. These results were consistent with recently published studies demonstrating that gene induction technology significantly induced AQP4 expression in vivo.^[26,27]^ In an additional study, gensenoide Rb1 treatment or overexpression of AQP4 significantly attenuated the OGD/R-induced cellular membrane permeability, which further verified that gensenoide Rb1 prevents OGD/R-induced astrocyte edema via AQP4 upregulation.

Since there is currently a lack of drugs that can reduce cellular edema, glucocorticoids are the most commonly used drugs to alleviate acute spinal cord edema, and their mechanism of action may be related to the increase of AQP4 expression. However, their clinical application is limited because of their side effects, short action time, and poor efficacy.^[28]^ In addition, new drugs, such as sulforaphane, can directly reduce SCII through upregulation of AQP4 expression, but its side effects are more serious, which limit its clinical application.^[29]^ Therefore, the development of new drugs for the treatment of SCII, especially by reducing the edema of spinal cord cells, will have important effects on society.

Ginsenoside Rb1 monomer is a traditional Chinese medicine ginseng extract that has been used for the treatment of nervous system diseases and nerve injury.^[30,31]^ Our previous study indicated that ginsenoside Rb1 treatment obviously prevented SCII in a mouse model through upregulation of AQP4.^[24]^ Furthermore, here, we verified that ginsenoside Rb1 significantly attenuated the OGD/R-induced AQP4 decrease (Fig. 1) and the astrocyte water permeability rate increase (Fig. 3), suggesting that the antiedema activity of ginsenoside Rb1 in the SCII process might be via triggering AQP4 expression in astrocytes. Lv et al have reported that NGF can prevent AQP4-induced edema in rats.^[19]^ In addition, Jones et al have shown that BDNF, a member of the neurotrophin family, can protect certain neurons of the peripheral and central nervous systems.^[20]^ In the astrocyte OGD model, we found that both NGF and BDNF significantly decreased compared with the nontreatment control and that ginsenoside Rb1 treatment partially attenuated this downregulation (Fig. 4), suggesting that the antiedema activity of ginsenoside Rb1 may be via upregulation of NGF and BDNF, which trigger AQP4 expression and thus decrease cellular water permeability. Further experiments to verify this finding in vivo are necessary and are underway in our research group.

## 5 Conclusion

In an astrocyte OGD ischemia-reperfusion model, we first demonstrated that AQP4 expression was significantly decreased, the water permeability rate was dramatically increased, and NGF and BDNF expression were obviously reduced. The pivotal role of AQP4 in cellular edema prevention was then verified by *AQP4* gene knockdown by siRNA transfection, which significantly increased the cellular water permeability rate. Thus, ginsenoside Rb1 treatment clearly attenuated the OGD-induced AQP4, NGF, and BDNF downregulation and water permeability.

## Author contributions

**Data curation:** Ya-Nan Li, Zhong-Wen Gao, Ran Li, Yun-Feng Zhang, Fei Huang.

**Formal analysis:** Ya-Nan Li, Zhong-Wen Gao, Fei Huang.

**Funding acquisition:** Fei Huang.

**Investigation:** Ya-Nan Li, Ran Li, Qing-San Zhu, Fei Huang.

**Methodology:** Ya-Nan Li, Zhong-Wen Gao, Ran Li, Yun-Feng Zhang, Qing-San Zhu, Fei Huang.

**Project administration:** Ya-Nan Li, Zhong-Wen Gao, Qing-San Zhu.

**Resources:** Fei Huang.

**Software:** Yun-Feng Zhang, Qing-San Zhu, Fei Huang.

**Supervision:** Ya-Nan Li, Ran Li, Qing-San Zhu, Fei Huang.

**Validation:** Yun-Feng Zhang, Fei Huang.

**Visualization:** Zhong-Wen Gao, Fei Huang.

**Writing – original draft:** Ya-Nan Li, Zhong-Wen Gao, Ran Li, Yun-Feng Zhang, Fei Huang.

**Writing – review and editing:** Ya-Nan Li, Fei Huang.

## References

[1] Smith PD Puskas F Meng X The evolution of chemokine release supports a bimodal mechanism of spinal cord ischemia and reperfusion injury. Circulation 2012;126:S110 7. 22965970 10.1161/CIRCULATIONAHA.111.080275

[2] Zhang Z Zhang G Sun Y Tetramethylpyrazine nitrone, a multifunctional neuroprotective agent for ischemic stroke therapy. Sci Rep 2016;6:37148. 27841332 10.1038/srep37148 PMC5107909

[3] Ikarashi N Kon R Sugiyama K Aquaporins in the colon as a new therapeutic target in diarrhea and constipation. Int J Mol Sci 2016;17:E1172. 27447626 10.3390/ijms17071172 PMC4964543

[4] Michalek K Aquaglyceroporins in the kidney: present state of knowledge and prospects. J Physiol Pharmacol 2016;67:185 93. 27226178

[5] Filippidis AS Kalani MY Rekate HL Hydrocephalus and aquaporins: the role of aquaporin-4. Acta Neurochir Suppl 2012;113:55 8. 22116424 10.1007/978-3-7091-0923-6_12

[6] Hubbard JA Szu JI Binder DK The role of aquaporin-4 in synaptic plasticity, memory and disease. Brain Res Bull 2018;136:118 29. 28274814 10.1016/j.brainresbull.2017.02.011

[7] Wolburg H Wolburg-Buchholz K Fallier-Becker P Structure and functions of aquaporin-4-based orthogonal arrays of particles. Int Rev Cell Mol Biol 2011;287:1 41. 21414585 10.1016/B978-0-12-386043-9.00001-3

[8] Tang G Yang GY Aquaporin-4: a potential therapeutic target for cerebral edema. Int J Mol Sci 2016;17:E1413. 27690011 10.3390/ijms17101413 PMC5085613

[9] Li W Yan MH Liu Y Ginsenoside Rg5 ameliorates cisplatin-induced nephrotoxicity in mice through inhibition of inflammation, oxidative stress, and apoptosis. Nutrients 2016;8: E566. 10.3390/nu8090566 PMC5037551 27649238

[10] Wang B He L Cui B Protection of ginsenoside Rg1 on central nerve cell damage and the influence on neuron apoptosis. Pak J Pharm Sci 2014;27 (6 Suppl):2035 40. 25410069

[11] Sharma HS Muresanu DF Lafuente JV Nanoparticles exacerbate both ubiquitin and heat shock protein expressions in spinal cord injury: neuroprotective effects of the proteasome inhibitor carfilzomib and the antioxidant compound H-290/51. Mol Neurobiol 2015;52:882 98. 26126513 10.1007/s12035-015-9297-9

[12] Bracken MB Steroids for acute spinal cord injury. Cochrane Database Syst Rev 2012;1:CD001046. 22258943 10.1002/14651858.CD001046.pub2 PMC6513405

[13] Schnell L Hunanyan AS Bowers WJ Combined delivery of Nogo-A antibody, neurotrophin-3 and the NMDA-NR2d subunit establishes a functional ‘detour’ in the hemisected spinal cord. Eur J Neurosci 2011;34:1256 67. 21995852 10.1111/j.1460-9568.2011.07862.x PMC3195885

[14] Attele AS Wu JA Yuan CS Ginseng pharmacology: multiple constituents and multiple actions. Biochem Pharmacol 1999;58:1685 93. 10571242 10.1016/s0006-2952(99)00212-9

[15] Song ZP Xiong BR Guan XH Minocycline attenuates bone cancer pain in rats by inhibiting NF-(B in spinal astrocytes. Acta Pharmacol Sin 2016;37:753 62. 27157092 10.1038/aps.2016.1 PMC4954763

[16] Lin SP Ye S Long Y Circular RNA expression alterations are involved in OGD/R-induced neuron injury. Biochem Biophys Res Commun 2016;471:52 6. 26845359 10.1016/j.bbrc.2016.01.183

[17] Livak KJ Schmittgen TD Analysis of relative gene expression data using real-time quantitative PCR and the 2(-Delta Delta C(T)) method. Methods 2001;25:402 8. 11846609 10.1006/meth.2001.1262

[18] Melamud L Fernández JM Rivarola V Neuromyelitis optica immunoglobulin G present in sera from neuromyelitis optica patients affects aquaporin-4 expression and water permeability of the astrocyte plasma membrane. J Neurosci Res 2012;90:1240 8. 22354518 10.1002/jnr.22822

[19] Lv Q Fan X Xu G Intranasal delivery of nerve growth factor attenuates aquaporins-4-induced edema following traumatic brain injury in rats. Brain Res 2013;1493:80 9. 23183041 10.1016/j.brainres.2012.11.028

[20] Jones KR Reichardt LF Molecular cloning of a human gene that is a member of the nerve growth factor family. Proc Natl Acad Sci U S A 1990;87:8060 4. 2236018 10.1073/pnas.87.20.8060 PMC54892

[21] Sun L Li M Ma X Inhibition of HMGB1 reduces rat spinal cord astrocytic swelling and AQP4 expression after oxygen-glucose deprivation and reoxygenation via TLR4 and NF-(B signaling in an IL-6-dependent manner. J Neuroinflammation 2017;14:231. 29178911 10.1186/s12974-017-1008-1 PMC5702193

[22] Wang YF Gu YT Xu WB Temporary loss of perivascular aquaporin-4 in white matter after the spinal cord ischemic injury of rats. Neuroreport 2009;20:145 9. 19151597 10.1097/WNR.0b013e32831c6c44

[23] Liu Y Pan L Jiang A Hydrogen sulfide upregulated IncRNA CasC7 to reduce neuronal cell apoptosis in spinal cordischemia-reperfusion injury rat. Biomed Pharmacother 2018;98:856 62. 29571256 10.1016/j.biopha.2017.12.079

[24] Huang F Li YN Yin F Ginsenoside Rb1 inhibits neuronal apoptosis and damage, enhances spinal aquaporin 4 expression and improves neurological deficits in rats with spinal cord ischemia-reperfusion injury. Mol Med Rep 2015;11:3565 72. 25573543 10.3892/mmr.2015.3162

[25] Zheng Y Wang L Chen M Upregulation of miR-130b protects against cerebral ischemic injury by targeting water channel protein aquaporin 4 (AQP4). Am J Transl Res 2017;9:3452 61. 28804561 PMC5527259

[26] Papadopoulos MC Verkman AS Aquaporin 4 and neuromyelitis optica. Lancet Neurol 2012;11:535 44. 22608667 10.1016/S1474-4422(12)70133-3 PMC3678971

[27] Nesic O Guest JD Zivadinovic D Aquaporins in spinal cord injury: the janus face of aquaporin 4. Neuroscience 2010;168:1019 35. 20109536 10.1016/j.neuroscience.2010.01.037 PMC2885549

[28] Fujihara K Treatment of neuromyelitis optica. Nihon Rinsho Meneki Gakkai Kaishi 2012;35:129 35. 22576570 10.2177/jsci.35.129

[29] Mao L Wang HD Pan H Sulphoraphane enhances aquaporin-4 expression and decreases spinal cord oedema following spinal cord injury. Brain Inj 2011;25:300 6. 21280976 10.3109/02699052.2010.542432

[30] Wang YZ Xu Q Wu W Brain transport profiles of ginsenoside Rb1 by glucose transporter 1: in vitro and in vivo. Front Pharmacol 2018;9:398. 29725302 10.3389/fphar.2018.00398 PMC5917093

[31] Zhao J Lu S Yu H Baicalin and ginsenoside Rb1 promote the proliferation and differentiation of neural stem cells in Alzheimer's disease model rats. Brain Res 2018;1678:187 94. 29038007 10.1016/j.brainres.2017.10.003

